# The Marri Gudjaga project: a study protocol for a randomised control trial using Aboriginal peer support workers to promote breastfeeding of Aboriginal babies

**DOI:** 10.1186/s12889-023-15558-2

**Published:** 2023-05-04

**Authors:** Rebecca Thorne, Rowena Ivers, Michelle Dickson, Karen Charlton, Lisa Jackson Pulver, Christine Catling, Michael Dibley, Simon Eckermann, Shahla Meedya, Miranda Buck, Patrick Kelly, Elizabeth Best, Melanie Briggs, Joan Taniane

**Affiliations:** 1 University of Wollongong, Wollongong, Australia; 2 University of Sydney, Sydney, Australia; 3 University of Technology Sydney, Sydney, Australia; 4 Latrobe University, Melbourne, Australia; 5 NSW Ministry of Health, NSW, Australia; 6 Waminda — South Coast Women’s Health & Welfare Aboriginal Corporation, Nowra, Australia; 7 Australian Catholic University, Sydney, Australia

**Keywords:** Breastfeeding, Aboriginal, Peer support, Breastfeeding initiation

## Abstract

**Background:**

Breastfeeding protects against a range of conditions in the infant, including sudden infant death syndrome (SIDS), diarrhoea, respiratory infections and middle ear infections [1, 2]. The World Health Organization (WHO) recommends exclusive breastfeeding until six months of age, with continued breastfeeding recommended for at least two years and other complementary nutritious foods [3]. The 2017-18 National Health Survey (NHS) and 2018-19 National Aboriginal and Torres Strait Islander Health Survey (NATSIHS) reported that the proportion of breastfeeding in Aboriginal and Torres Strait Islander infants (0–2 years) were less than half that of non-Indigenous infants (21.2% vs. 45%, respectively)[4]. There is a lack of research on interventions supporting Aboriginal women to breastfeed, identifying an evaluation gap related to peer support interventions to encourage exclusive breastfeeding in Aboriginal women.

**Methods:**

We will evaluate the effect of scheduled breastfeeding peer support for and by Aboriginal women, on breastfeeding initiation and the prevalence of exclusive breastfeeding. This MRFF (Medical Research Future Fund) funded project is designed as a single-blinded cluster randomised controlled trial recruiting six sites across New South Wales, Australia, with three sites being randomised to employ a peer support worker or undertaking standard care. Forty pregnant women will be recruited each year from each of the six sites and will be surveyed during pregnancy, at six weeks, four and six months postnatally with a single text message at 12 months to ascertain breastfeeding rates. In-depth interviews via an Indigenous style of conversation and storytelling called ‘Yarning’ will be completed at pre- and post-intervention with five randomly recruited community members and five health professionals at each site” [5]. Yarns will be audio recorded, transcribed, coded and thematic analysis undertaken. Health economic analysis will be completed to assess the health system incremental cost and effects of the breastfeeding intervention relative to usual care.

**Discussion:**

Evidence will be given on the effectiveness of Aboriginal peer support workers to promote the initiation and continuation of breastfeeding of Aboriginal babies. The findings of this study will provide evidence of effectiveness and cost-effectiveness of including peer support workers in postnatal care to promote breastfeeding practices.

**Trial Registration:**

ACTRN12622001208796 The impact of breastfeeding peer support on nutrition of Aboriginal infants

**Supplementary Information:**

The online version contains supplementary material available at 10.1186/s12889-023-15558-2.

## Background

Breastfeeding protects against a range of conditions in the infant, including sudden infant death syndrome (SIDS), diarrhoea, respiratory infections and middle ear infections [1, 2]. There are also later benefits associated with being breastfed, for both children and adults, such as a lower risk of developing obesity in childhood, diabetes in adulthood and other later cardiometabolic disorders [2, 6]. Breastfeeding also benefits women, including more rapid weight loss after birth, and reduced risk of breast and ovarian cancer in the premenopausal phase of life [2, 7].The World Health Organization (WHO) recommends exclusive breastfeeding until six months of age, with continued breastfeeding recommended for at least two years and providing of other complementary nutritious foods [3]. Similarly, Australian National Health and Medical Research Council (NHMRC) guidelines recommend that infants are exclusively breastfed until around six months of age, at which age solid foods are introduced, and recommends that breastfeeding is continued until 12 months of age and beyond [2].

Data collected from the 2017-18 National Health Survey (NHS) and 2018-19 National Aboriginal and Torres Strait Islander Health Survey (NATSIHS) in Australia reported that the proportion of Aboriginal and Torres Strait Islander infants (0–2 years) currently being breastfed was half that of non-Indigenous infants (21.2% vs. 45%, respectively)[4]. Data also showed that Aboriginal And Torres Strait Islander infants had higher rates of never being breastfed (13.0% vs. 7.7%, respectively)[4].

The 2012-3 Australian Aboriginal and Torres Strait Islander Health Survey reported that Aboriginal infants aged 0–3 months in New South Wales (NSW), Australia were exposed to the lowest rates of breastfeeding (12%) compared to other Aboriginal or non-Aboriginal infants in other states [8].

Aboriginal and Torres Strait Islander infants are also more likely to be weaned onto solid foods before six months of age; 45% of infants received soft/ solid food by four months, compared with 28% of non-Indigenous infants [9]. Early weaning increases the risk of gastroenteritis, [2] particularly in environments where hygiene practices required for sterilising bottles may not be easily achieved or maintained, as in the case of overcrowding.

In addition to health implications, commercial infant formula creates an additional economic burden to families, compared to breastfeeding [9, 10]. The total value of breastfeeding to the community makes it one of the most cost-effective primary prevention measures available [2, 11, 12]. A number of studies have calculated the potential economic benefits of breastfeeding. A United States analysis showed that if 90% of families could exclusively breastfeed for six months, the country would save $13 billion per year in healthcare costs and prevent an excess 911 deaths per year, nearly all of whom would be infants [13]. An Australian study has estimated that the hospital-related cost of premature weaning for four conditions (gastrointestinal illness, respiratory illness, eczema and necrotising enterocolitis) as AU$60–120 million per annum [14]. In Canada, McIsaac [15] calculated the proportion of three infectious outcomes and one mortality outcome that could be prevented in infancy by breastfeeding for First Nations Métis and Inuit populations. Between 5.1% and 10.6% of otitis media, 24.3 − 41.4% of gastrointestinal infection, 13.8 − 26.1% of hospitalisations from lower respiratory tract infections, and 12.9 − 24.6% of sudden infant deaths could be prevented in Aboriginal and Torres Strait Islander infants if they received any breastfeeding. No such calculations have been carried out for Aboriginal or Torres Strait Islander Australians.

### Interventions

There is good evidence for including peer support interventions to support women’s decision to initiate breastfeeding and continue for a longer period of time [16, 17]. A Cochrane review of interventions to increase breastfeeding in infants born at term; had more than 83,246 mother-infant pairs from 73 studies that contributed data (58 individually-randomised trials and 15 cluster-randomised trials) [18]. Overall, interventions increased exclusive breastfeeding rates and duration. Several factors that contributed to the successful interventions that encouraged exclusive breastfeeding included: advice offered as part of routine care by trained personnel during antenatal or postnatal care; ongoing scheduled visits; and advice that was tailored to the setting and needs of the specific population group. Other characteristics of successful strategies included interventions delivered with a face-to-face component, lay/ peer support, and a specific schedule of four to eight contacts [18]. The evidence [18] identified that mixed mode interventions, combinations of face-to face and other media support, were effective when they start from pregnancy and continues to postnatal period [17]. Support was more likely to be effective in settings that had already high breastfeeding initiation rates. One of the studies included in the Cochrane review, a cluster randomised controlled trial of motivational interviewing to extend the duration of lactation in 300 women in regional NSW [19], showed a greater prevalence of exclusive breastfeeding in the intervention group at four months (adjusted OR 1.88; 95%CI 1.01–3.50; p = 0.047). A rapid evidence review to inform the Australian Breastfeeding Strategy notes that evidence from the most recent comprehensive systematic reviews highlights that additional education or support for mothers of any kind within the healthcare system, compared with usual care, increases the likelihood of breastfeeding by 10–30%. Interventions that included peer counselling were more effective than those without peer counselling [10]. Studies also confirmed that face-to-face care is more effective than other communication, for example printed materials. Nevertheless, interventions involving technology as an adjunct to other postnatal support have recently been found to increase exclusive breastfeeding [10, 20].

There is almost no research on interventions to support Aboriginal and Torres Strait Islander women to breastfeed, identifying an evaluation gap related to peer support interventions to encourage exclusive breastfeeding in Aboriginal and Torres Strait Islander women. There is a need for translational research and for capacity-building based on the effectiveness and cost-effectiveness of community-based social mobilisation and breastfeeding education activities. Our study addresses that evidence gap.

Successful breastfeeding interventions conducted in Aboriginal and Torres Strait Islander populations in other countries include: peer counselling support, multifaceted components that are culturally appropriate; inclusion of social marketing techniques; provision of more baby-friendly healthcare services; inclusion of individual education and support; and targeting of family members including the grandmother [10]. In Australian, a review of current evidence points towards models of care that provide continuity of care, delivered by Aboriginal and Torres Strait Islander community-controlled organisations, Aboriginal and Torres Strait Islander health professionals and employed peer workers as most effective [10].

There is minimal published research internationally for the cost of peer support, or even professional support, for lactation support. In one trial, similar to our proposal, the FEeding Support Team (FEST), conducted a randomised, controlled feasibility trial of proactive and reactive telephone support for breastfeeding women living in disadvantaged areas in Scotland, with follow-up at six weeks. The incremental cost of providing proactive calls was £87 per additional woman breastfeeding and £91 per additional woman exclusively breastfeeding at 6–8 weeks [21].

The Australian Breastfeeding Association (ABA) is Australia’s most prominent non-government organisation supporting breastfeeding families. They developed and first delivered a training program for community breastfeeding mentors in Aboriginal communities in in NSW in 2019 and included Aboriginal workers in maternal and child health programs and community members. We plan to use this training model to train workers. We are not aware of paid positions for Aboriginal peer workers that continue beyond the six-week postnatal period. Our review of online resources to support Aboriginal women to breastfeed revealed few resources that are culturally appropriate to support Aboriginal families to breastfeed[22] but included some websites, pamphlets and video resources[22] [23].

### Pilot work

In 2016, we initiated a lactation support group in response to low breastfeeding rates in one location in regional NSW, Australia. The support group, held at a local cultural centre, was run by a child health nurse and an Aboriginal Health Worker and included an Aboriginal peer support volunteer. Following zero attendance at this lactation support group, we undertook a small qualitative research project (unpublished) to interview Aboriginal women with infants about the facilitators and barriers to breastfeeding. Women reported negative and positive feedback about their experiences related to breastfeeding advice that had been provided by health professionals. Some women found it difficult to access advice when they were experiencing difficulties with breastfeeding, including after-hours access for support. Women did not state that they were concerned about breastfeeding in public or about the effects of breastfeeding on their appearance. One participant noted that access to a face-to-face online resource was useful. However, most participants were unaware of this service, or reported that they could not afford to access a lactation consultant.

### Objectives

This project aims to improve the nutrition of Aboriginal and Torres Strait Islander infants.

Our objective is to evaluate the effect of scheduled breastfeeding peer support for and by Aboriginal women, on breastfeeding initiation and the prevalence of exclusive breastfeeding at six weeks, four months and six months post–birth. The intervention will utilise face-to-face peer support provided for and by Aboriginal women and employ innovative aspects such as social media, video and telephone calls by Aboriginal peer workers for six months postnatally.

## Methods/design

### Overview

**Fig. 1:**
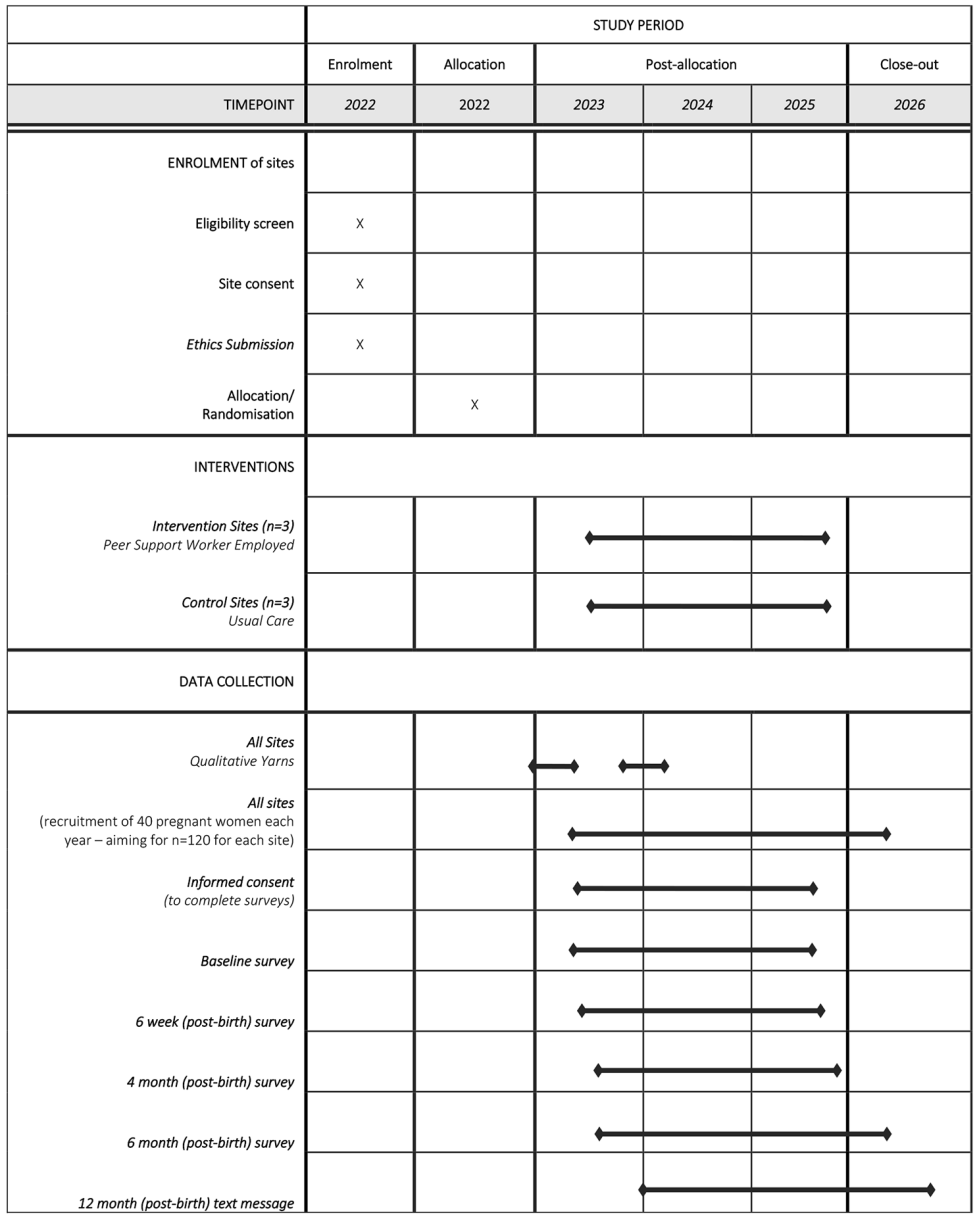
Marri Gudjaga — SPIRIT schedule of enrolment, intervention and data collection

The project design is a single-blinded cluster randomised controlled trial of an Aboriginal peer support worker to support the initiation and maintenance of breastfeeding over the first six months of life. Other research objectives include the evaluation of the role of these workers via qualitative interviews/yarns with community and health professionals, and development and evaluation of video resources for community members. The schedule of enrolment, intervention and data collection can be found in Fig. 1.

### RCT

#### Sites/Setting

The RCT will include six Aboriginal maternal and child health services in NSW (NSW Aboriginal Maternal and Infant Health Services, and Aboriginal Community Controlled Health Services).

NSW was chosen as the geographical area to sample because Aboriginal women in NSW have the lowest initiation rates for breastfeeding and the highest decline in exclusive breastfeeding rates in the country [9]. In NSW, much of the antenatal care for Aboriginal women is delivered through Aboriginal Community Controlled Health Organisations (ACCHOs) and through Aboriginal Maternal and Infant Health Service (AMIHS) via NSW Health. Some Aboriginal women access mainstream general practices, hospital antenatal clinics and private obstetricians. For the purpose of this study, we anticipate recruiting women attending antenatal care services that target Aboriginal communities specifically, including the maternal and child health services within the AMIHs network, which includes 40 services, as well as an independent community-controlled maternal and child health service. These services provide care to Aboriginal women, both antenatally and postnatally.

Peer workers will be recruited at intervention sites, including via Aboriginal community networks. The workers will be employed by the University of Wollongong (UOW) but work within the partner organisations under a Memorandum of Understanding (prepared by the UOW legal team in conjunction with individual Local Health Districts or organisations), under the supervision of the UOW team.

Participating sites will be offered compensation for involvement in the trial including funds for infrastructure support for the peer worker (including funding for fuel/ transport costs, and office infrastructure such as computer and phone). Sites are also offered funding to support workers including the peer worker and 1–2 other health care workers to attend annual training workshops/ seminars.

#### Sample size

We plan to recruit a total of 720 participants (120 at each site), allowing for a predicted 15% drop out at 6 months post-birth, resulting in 612 participants for analyses.

A similar intervention, the RUBY Study (Forster et al., 2017) was able to follow-up 88% of participants so we believe a 15% drop-out rate is likely conservative.

#### Participant inclusion criteria

Aboriginal or Torres Strait Islander women or non-Aboriginal women who have an Aboriginal or Torres Strait Islander infant that they are breastfeeding will be included. Participants (mothers) will be over 14 years of age. Those who are aged 14–17 will need to obtain parental/guardian permission for involvement.

#### Participant recruitment

Recruitment of participants will be conducted from 28 weeks gestation and survey collection will occur from 32 weeks gestation with care to recruit young and marginalised women to prevent under-sampling.

Staff at the participating sites will offer the woman a show bag containing information about the project, giveaways (as determined by staff at the site, funded by the project), and the Participant Information Statement PIS). Staff will ask verbal permission from the participant to pass on their mobile phone number (landline and/or email) to the research team so they may to be contacted regarding the study.

Staff at the sites will record the potential participant’s name on a log that contains the woman’s name, mobile number, and Estimated Due Date (EDD). This information will be scanned by the staff member and emailed or faxed to the research team, weekly.

All participants will be provided with a $50 gift card at the six-week interview (6 weeks post-birth) as an incentive for participating in the project. Those who do not have a mobile phone can be provided with one as part of the project.

#### Randomisation of Sites

The study design involves simple cluster randomisation so that sites will be randomised and stratified according to rurality (using the Modified Monash Model (MMM)). We recruited interested maternal and child health services from all over NSW with more than 50 births per year. Stratified randomisation is key to avoiding potential for unbalanced factors between arms to maintain comparability or control for random variability. Randomisation will stratify by the MMM – MM1 (major city) through to MM3 (large rural towns) using a computer-based program by a biostatistician.

#### Consent

Participants will be provided with written information about the project - the Participant Information Sheet (PIS) - contained in the show bag offered by site staff. Consent will be provided over the phone (for phone survey collection) or via implied consent (emailed survey or survey link sent by SMS). For participants between 14 and 17 years old, additional consent will be sought from a parent or guardian (audio-recorded verbal consent at the commencement of the project).

#### Data collection

**Fig. 2:**
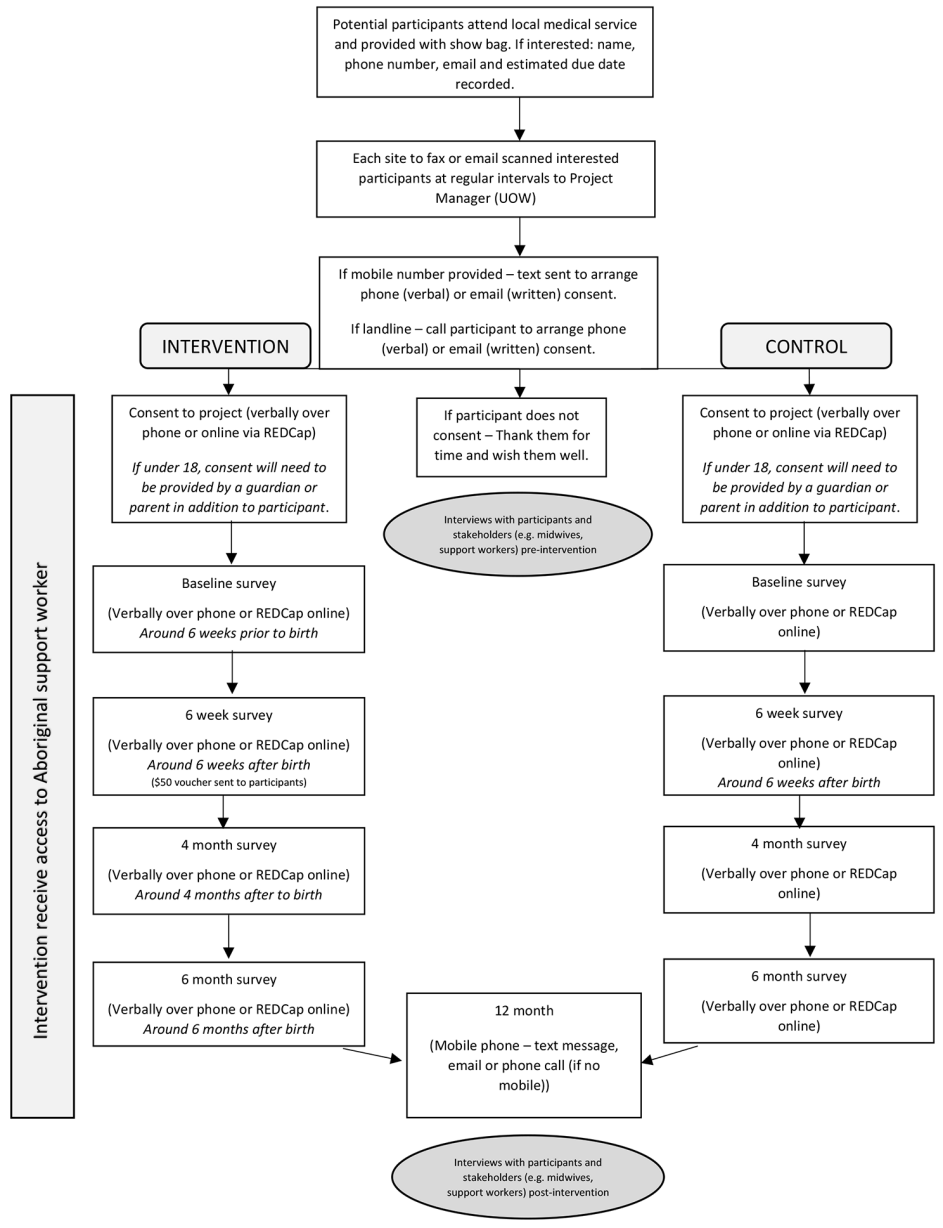
RCT Flowchart

A research assistant or associate research fellow will collect data. The research team will call potential participants that have provided their contact details. They will again be verbally given information about the project, from the PIS and will be asked to provide verbal consent. Surveys will be collected at these time-points (see Fig. 2):

- Baseline (32–40 weeks of gestation).
- 6 weeks post-birth (after which a $50 voucher will be sent to participant).
- 4 months post-birth.
- 6 months post-birth.
- 12 months post-birth (SMS message).

If participants are not contactable by telephone after three attempts, a link to the survey will be emailed to them or sent via SMS (to a maximum of three times over three weeks). Permission will also be requested for the maternal and child health team to inform peer workers when the women have given birth to their babies, in order to ensure early access to the intervention.

#### Intervention

##### Peer support workers will be employed three days a week for three years in three communities

We plan an **evidence-based, culturally appropriate and best practice intervention** designed with the aim to improve the initiation of breastfeeding and support the continuation of exclusive breastfeeding to best practice standards. Following the systematic review of the evidence the intervention will include:

Employment of Aboriginal breastfeeding peer support workers, who community mentor training, provided through the ABA. Trained Aboriginal peer support workers will then be hosted by maternal and child services in three sites randomised to receive the intervention.

The intervention group will receive scheduled breastfeeding Aboriginal peer breastfeeding support at an antenatal visit, then again within the first week after birth and then support over the following 6 months. The peer workers will provide support such as weekly telephone calls and/or SMS text messages for 6 weeks after birth, along with face-to-face visits where possible (for example clinic visits, mothers’ group or home visits when accompanying other staff). As appropriate, video-chats will be included, as part of a program of monthly Aboriginal peer worker video-chats (via smart-phone video) about breastfeeding. Opportunistic support will also be provided for emerging issues, for example nipple pain. If required, breastfeeding technical support will be sought by the Aboriginal peer support workers from experienced midwives, Aboriginal Health Workers (AHWs), Aboriginal Health Practitioners, ABA counsellors or lactation consultants, or women would be referred to these health care professionals for medical support (such as support for mastitis). Our team will offer cultural safety training for ABA peer counsellors and lactation consultants where appropriate, to ensure they can provide local, culturally appropriate/informed technical support for the Aboriginal peer workers and women.

#### Control

Normal/usual antenatal and postnatal care in three communities.

All sites will receive usual lactation support, including advice from midwives and AHW in antenatal programs, early childhood nurses and AHW, advice from midwives, junior doctors and paediatricians in hospital maternity wards, and from lactation consultants based in hospitals. Other lactation support is provided by general practitioners (for example in Aboriginal medical services), child health nurses and community lactation consultants on demand or during routine postnatal checks and immunisation reviews. Some Aboriginal women also may access private lactation consultants or access the ABA hotline.

#### Analysis

Data from telephone/email surveys at baseline prior to birth, 6 weeks, 4 months and 6 months will be entered into a matrix in REDCap ©, an electronic data collection program by the research team. This data will be collated into Excel, cleaned and analysed, with supervision by the biostatistician. Data will be coded, Statistician and Health Economist will remain blinded throughout the length of the study.

- Peer worker interactions with participants will be logged, along with notes about factors which might affect uptake (i.e. participants not available to respond, other priorities such as funerals) so as to undertake process analysis, in addition to outcome analysis. This will be scanned/emailed to the research team for analysis.
- Interactions with participants will be logged by peer workers (on paper/electronic, stored in a locked secure environment at the site, then emailed or scanned to the research team and entered into REDCap©) to ensure scheduled visits had occurred, and to record any issues that could be incorporated into a process analysis (e.g. participant did not respond, participant had moved).
- We will use ‘journal’ reflective practice data for analysis of Aboriginal peer workers’ interactions [24].

We plan to recruit at least 360 participants and 306 participants completing 6 weeks in both of group 1 (intervention) and group 2 (control), with three sites in each group (120 participants and 102 completing 6 weeks per site assuming 15% drop out) providing 80.1% power to detect a difference between the group proportions of 0.1500. The proportion in group 1 (the treatment group) is assumed to be 0.2500 under the null hypothesis and 0.4000 under the alternative hypothesis. The proportion in group 2 (the control group) is 0.2500. The test statistic used is the two-sided Z-Test (Pooled), the intra-cluster correlation is 0.0100 and the significance level of the test is 0.050.

At 6 weeks we expect a prevalence of exclusive breastfeeding to be 25% in the control group, based on breastfeeding prevalence of 26% seen at 6 weeks in the Gudaga study (Craig et al., 2011) while if effective we estimate prevalence of exclusive breastfeeding in the intervention group to be 40% or more. A Cochrane review by McFadden et al. [18] reported women in intervention groups were less likely to stop exclusive breastfeeding by 6 weeks (RR 0.79, 95% CI 0.71 to 0.89)., which applied to a base rate of 75% stopping breastfeeding leads to 15% higher absolute breastfeeding rates at 6 weeks (40% vs. 25%). However, we consider the current study intervention to be more intense than other studies included in that meta-analysis.

Elliot-Rudder [19] conducted a cluster RCT where health professionals were trained in motivational interviewing to support women to continue breastfeeding. In a sample of 330 women, with the outcome of exclusive breastfeeding at 4 months (OR 1.88, 95% CI 1.01–3.6, p = 0.047), this study was able to demonstrate effectiveness of the intervention.

We plan to use survival analysis to assess one of our secondary outcomes (using another outcome, days of exclusive breastfeeding).

We will recruit approximately 120 mother – babies in each of six sites (aiming for 40 per year for 3 years, given approximately 80–100 births per year in each site).

Primary outcomes: (a) Exclusive breastfeeding at 6 weeks (24 h recall telephone questionnaire or via survey email). (b) Exclusive breastfeeding at 4 months (24 h recall telephone or survey email) (c) Exclusive breastfeeding at 6 months (24 h recall telephone or survey email).

Secondary outcomes: (a) Initiation of breastfeeding within first 24 h (self – report, 6 week survey), (b) Days of exclusive breastfeeding (self-report) (c) Days of any breastfeeding (self-report) (d) Age at introduction of expressed breastmilk (self-report). (e) Age introduction of solid/semi-solid food (self-report) (f) Age at introduction of other breastmilk substitutes (self-report) (g) Reason for cessation of breastfeeding including contraindications to breastfeeding (self-report) (h) Hospitalisation in first 6 months of life (self – report at six months) (i) Otitis media requiring antibiotics in first six months of life (self – report at six months). (j) Breastfeeding at 12 months (SMS – yes/ no).

Demographic and risk data including Estimated Due Date (EDD), age, pre/post-pregnancy weight/height (BMI) (at baseline, and weight at six months, self-reported), smoking status (at baseline and 6 months, self-reported), educational level, employment after birth and socioeconomic status (as obesity, low education, low socioeconomic status and returning to work are associated with decreased breastfeeding). We will also ask about previous breastfeeding experience and parity.

### Qualitative interviews

Interviews/Yarns with community and health professionals will be completed before and after the intervention from all six sites participating in the RCT. Qualitative evaluation will be conducted using Yarning methodology [5]. Yarning is considered “a way to safely engage with participants to explore research questions relating to the topic of the study” [25].

We anticipate the recruitment of 60 participants in the qualitative aspect of the study to achieve saturation of findings (ten per site – 5 community members and 5 stakeholders).

Yarns would be conducted prior to the intervention (RCT) and after 6–12 months of the intervention period, in order to identify barriers and facilitators for breastfeeding, and to assess acceptability of the new peer support breastfeeding intervention.

#### Sites

Participants for yarns/interviews will be recruited from the six sites participating in the RCT.

#### Sample size

60–66 participants to reach saturation of findings.

#### Recruitment

Members of the operation team will liaise with site staff in regarding the recruitment of community members (aged over 14 years, and if under 18, parental written or verbal consent required) and stakeholder/staffs (aged over 18) working within the service.

#### Consent

Community participants would be given a participant information sheet (PIS) and service providers would be given a separate PIS. Written consent will be required. All information would be also delivered verbally by the research team. Community participants would be asked for written consent (or recorded verbal consent where this was impossible). Service providers would be asked for written consent (or recorded verbal consent where this was not possible). All participants will be given a study ID to de-identify personal information in transcription and analysis. We will collect basic demographic details from participants (age, gender, role or relationship to participant).

Yarns will be conducted with the following:

##### Community members

participants, partners and aunties/grannies (10–15 before intervention and 10–15 after peer worker in place).

##### Health professionals

we plan to Yarn with AHWs, Aboriginal Health Practitioners, child health nurses and midwives, general practitioners, ABA volunteers and lactation consultants. (10–15 before intervention and 10–15 after peer worker is in place) as well as interviews with the six peer workers.

#### Data Collection

Yarns will be conducted either face to face as an interview or focus group, which will be digitally audio-recorded or using online video/phone interview where face to face interaction is not possible (also audio-recorded). These would be transcribed by an Australian transcription service. Interviews/ focus groups would be conducted using a Yarning guide. Community participants will be offered a $50 voucher for participating in the interview or focus group.

#### Analysis

Transcriptions will be analysed, using Yarning methodologies [5] and grounded theory to develop themes of barriers and facilitators to breastfeeding with and without access to a peer worker. We also plan to use the theory of planned behaviour [26] to consider factors contributing to breastfeeding choices and interaction with peer support worker [27].

### Video Development

This project also involves development of educational videos to support breastfeeding. We plan to evaluate these resources once developed through small focus groups or interviews (when shown in a group) using Yarning methodology. Yarns will be conducted either in face to face as an interview or focus group (from an interview guide), which will be digitally audio - recorded or using online video intervention where face to face interaction is not possible (also audio-recorded). These would be transcribed by an Australian transcription service.

#### Recruitment

We plan to recruit community members (10–15) and service providers (10–15) to evaluate the videos.

#### Consent

Community participants will be given a PIS and service providers would be given a separate PIS. Written consent will be required. All information would be also delivered verbally by the research team. Community participants would be asked for written consent (or recorded verbal consent where this was not possible). Service providers would be asked for written consent (or recorded verbal consent where this was not possible). Community participants will be offered a $50 voucher for their participation. All participants will be given a study ID to de-identify personal information in transcription and analysis. We will collect basic demographic details from participants (age, gender, role or relationship to participant).

#### Sample size

10–15 community members and 10–15 service providers which was chosen so as to reach saturation of findings.

#### Interviews

Interviews/ focus groups will be conducted using a Yarning guide.

#### Analysis

We plan to use an inductive thematic analysis to consider views on the resources developed.

### Data Management

#### Confidentiality, Privacy and Data Integrity

The consent process will ensure that data collected about participants is stored to preserve confidentiality according to legal standards. Data will be collected onto an online secure database, REDCap*©* via a laptop computer, backed up daily. Audio and transcribed files will also be stored digitally, backed up daily on Cloudstor. All data would be de-identified prior to analysis, and publication.

A subgroup of investigators will form the Data Monitoring Committee which will meet annually to review data security and quality and accuracy of data collection.

#### Study records retention

All electronic data will be stored for 15 years following completion of the project, on the password locked University of Wollongong secure server (Research Data Storage and Management system, Cloudstor).

#### Data Handling and Management Responsibilities

Data will be stored securely in a password locked secure server (Cloudstor) at University of Wollongong. The ACCHS and AMIHS maternal and child health agencies involved will be given access to their site data and will be given feedback about the results of the project and asked for permission for public release, for example of reports, journal articles or conference presentations.

#### Trial governance

The project will appoint an Aboriginal Steering Committee, including investigators, midwife/ves, AHWs and consumers who will meet biannually. The purpose of the steering committee will be to advise the research team about appropriate service delivery models.

A subgroup of investigators will form the Data Monitoring Committee which will meet annually to review data security and quality and accuracy of data collection.

#### Dissemination of results

Our research team’s previous research work has focused on work that is developed and driven by the community and prioritises feedback to the community in addition to research outputs in the form of journal articles and conference presentations. Results would be offered (in the form of a culturally appropriate email summary) to all participants and will be fed back to community boards/managers verbally, in an easy-to-read report and infographic. Reporting would include specific recommendations on evidence for the economic benefits of this intervention. Findings will also be fed back to ACCHSs, state health services and the ABA where findings recommended changes to service delivery, for example evidence of effectiveness breastfeeding peer support workers.

### Health Economics

Health economic analysis will include assessment of incremental study and health system cost per additional breastfeeding woman, as assessed in the FEST study and more generally joint incremental effects and costs estimated under uncertainty [21]. Costs estimated will include direct costs of intervention staff time and overheads, their training with the Australian Breastfeeding Association, cost of car use in accessing populations, program consumables and $50 voucher for intervention participants completing 4 months follow up as observed in study and modelled downstream health system costs associated with effects of breastfeeding in each arm observed on study.

## Discussion

This study will be an opportunity to explore the peer support approach to promote initiation and continuation of breastfeeding in Aboriginal and Torres Strait Islanders. Potential outcomes could be trialling a new workforce model to include community roles within the health service.

## Electronic supplementary material

Below is the link to the electronic supplementary material.

Supplementary Material 1

Supplementary Material 2

Supplementary Material 3

Supplementary Material 4

Supplementary Material 5

Supplementary Material 6

Supplementary Material 7

## Data Availability

The data will not be part of a public repository but datasets may be accessed at completion of study pending ethics approval.

## References

[1] Khan J (2015). Timing of breastfeeding initiation and exclusivity of breastfeeding during the first month of life: effects on neonatal mortality and morbidity–a systematic review and meta-analysis. Matern Child Health J.

[2] National Health and Medical Research Council (2012). Literature Review: infant feeding guidelines.

[3] World Health Organisation. Breastfeeding. 2018 09/06/2021]; Available from: https://www.who.int/news-room/facts-in-pictures/detail/breastfeeding.

[4] Welfare, Australian Institute of Health and (2020). *AIHW and ABS analysis of National Aboriginal and Torres Strait Islander Health Survey 2018–19 and National Health Survey 2017–18*, A.I.o.H.a. Welfare, Editor.

[5] Bessarab D (2010). Ng’andu, *yarning about yarning as a legitimate method of indigenous research*. Int J Crit Indigenous Stud.

[6] McNamara BJ (2012). Early life influences on cardio-metabolic disease risk in aboriginal populations–what is the evidence? A systematic review of longitudinal and case-control studies. Int J Epidemiol.

[7] Kramer MS, Kakuma R. *Optimal duration of exclusive breastfeeding*.The Cochrane database of systematic reviews, 2012(8): p.CD003517. 10.1002/14651858.CD003517.pub2 PMC7154583 22895934

[8] Australian Bureau of Statistics (2013). Australian Aborginal and Torres Strait Islander Health Survey: first results.

[9] Australian Institute of Health and Welfare (2011). 2010 australian National Infant Feeding Survey: indicator results.

[10] Smith J, et al. Review of effectice strategies to promote breastfeeding: an evidence check rapid review brokered by the Sax Institute for the Department of Health. S. Institute, Editor.; 2018.

[11] Ageing, Australia Editor, House of Representatives Standing Committtee on Health and (2007). The best start: report on the inquiry into the health benefits of breastfeeding.

[12] Hansen K (2016). Breastfeeding: a smart investment in people and in economies. Lancet (London England).

[13] Bartick M, Reinhold A (2010). The burden of suboptimal breastfeeding in the United States: a pediatric cost analysis. Pediatrics.

[14] Smith JP, Thompson JF, Ellwood DA (2002). Hospital system costs of artificial infant feeding: estimates for the australian Capital Territory. Aust N Z J Public Health.

[15] McIsaac KE, Moineddin R, Matheson FI (2015). Breastfeeding as a means to prevent infant morbidity and mortality in Aboriginal Canadians: a population prevented fraction analysis. Can J Public Health.

[16] Matriano MG, Ivers R, Meedya S (2022). Factors that influence women’s decision on infant feeding: an integrative review. Women and birth: journal of the Australian College of Midwives.

[17] Meedya S, Fernandez R, Fahy K (2017). Effect of educational and support interventions on long-term breastfeeding rates in primiparous women: a systematic review and meta-analysis. JBI database of systematic reviews and implementation reports.

[18] McFadden A (2017). Support for healthy breastfeeding mothers with healthy term babies. Cochrane Database Syst Rev.

[19] Elliott-Rudder M et al. *Motivational interviewing improves exclusive breastfeeding in an Australian randomised controlled trial* Acta paediatrica (Oslo, Norway: 1992), 2014. 103(1): p. e11-e16. 10.1111/apa.12434 24117857

[20] Almohanna AA, Win KT, Meedya S (2020). Effectiveness of internet-based Electronic Technology Interventions on Breastfeeding Outcomes: systematic review. J Med Internet Res.

[21] Hoddinott P (2012). The FEeding support team (FEST) randomised, controlled feasibility trial of proactive and reactive telephone support for breastfeeding women living in disadvantaged areas. BMJ open.

[22] Hopkins M et al. *Review of online breastfeeding information for Aboriginal and Torres Strait Islander women* Women and birth: journal of the Australian College of Midwives, 2021. 34(4): p. 309–315. 10.1016/j.wombi.2020.06.012 32653396

[23] The Raising Children Network. Aboriginal and Torres Strait Islander parents. For professionals 2018 [cited 2021 09/06/2021]; Available from: https://raisingchildren.net.au/for-professionals/aboriginal-torres-strait-islanders-parents.

[24] Dickson ML (2017). Journal Conversations: building the Research Self-Efficacy of an Aboriginal Early Career Academic. Qualitative Rep.

[25] Kennedy M (2022). Decolonising qualitative research with respectful, reciprocal, and responsible research practice: a narrative review of the application of yarning method in qualitative Aboriginal and Torres Strait Islander health research. Int J Equity Health.

[26] Ajzen I (1991). The theory of planned behavior. Organ Behav Hum Decis Process.

[27] Wambach KA (2011). A randomized controlled trial of breastfeeding support and education for adolescent mothers. West J Nurs Res.

