# Supplementary material for: The Marri Gudjaga project: a study protocol for a randomised control trial using Aboriginal peer support workers to promote breastfeeding of Aboriginal babies
Source: BMC Public Health. 2023 May 4;23:823. doi: 10.1186/s12889-023-15558-2 (PMC10161673; doi:10.1186/s12889-023-15558-2)
Supplement: Supplementary file 3 — Supplementary Material 3 [file 12889_2023_15558_MOESM3_ESM.pdf]

UNIVERSITY OF WOLLONGONG AUSTRALIA

Marri gudjaga project

CONSENT FORM

I have been given information about the Marri gudjaga project, regarding the community views on the project, and had an opportunity to ask the research team any questions I may have about the research.

If I have any enquiries about the research, I can contact (Chief Investigator, Dr Rowena Ivers (rivers@uow.edu.au or (02) 4221 4341 or Project Manager, Miss Beck Thorne (marri-gudjaga@uow.edu.au or (02) 4221 5992).

By signing below I am indicating my consent to take part in this project.

I understand:

• That information I give will have my name removed (de-identified) and will be used to help the researchers gather information about what is the best way to support healthy nutrition for babies.

• That I am agreeing to participate in a yarn or yarning circle before and after the intervention to provide feedback to researchers.

• That the information may be used for a research report, journal article (for health professionals) or conference presentation, and I consent (agree) for it to be used in that manner.

SIGNED **DATE**

____ ____/____/____

Name (please print) ____

I consent to be video/audio recorded (only audio saved/stored) Yes/No

I would like to review a written form of my yarn Yes/No

I would like to receive an emailed copy of the report Yes/ No

Email (for those who would like a copy of the report) ____________________@_____________
