# Supplementary material for: The Marri Gudjaga project: a study protocol for a randomised control trial using Aboriginal peer support workers to promote breastfeeding of Aboriginal babies
Source: BMC Public Health. 2023 May 4;23:823. doi: 10.1186/s12889-023-15558-2 (PMC10161673; doi:10.1186/s12889-023-15558-2)
Supplement: Supplementary file 6 — Supplementary Material 6 [file 12889_2023_15558_MOESM6_ESM.docx]

## Marri gudjaga project

## CONSENT FORM

## I have been given information about the Marri gudjaga project and had an opportunity to ask the research team any questions I may have about the research.

As part of the project, videos have been developed to assist and support women in the community. We will be conducting yarning circles with community members to gain feedback on the videos.

If I have any enquiries about the research, I can contact (Chief Investigator, Dr Rowena Ivers ([rivers@uow.edu.au or (02)](mailto:rivers@uow.edu.au%20or%20(02)) 4221 4341 or Project Manager, Miss Beck Thorne ([marri-gudjaga@uow.edu.au](mailto:marri-gudjaga@uow.edu.au) or (02) 4221 5992).

By signing below I am indicating my consent to take part in this project.

I understand:

• That information I give will have my name removed (de-identified) and will be used to help the researchers gather information about what is the best way to support healthy nutrition for babies.

• That I am agreeing to participate in a yarning circles/yarn. I will be asked to review video aids and then provide feedback on the videos developed by researchers to assist women in the community.

• That the information may be used for a research report, journal article (for health professionals) or conference presentation, and I consent (agree) for it to be used in that manner.

**SIGNED DATE**

­­­­­­­­­­­­­­­­­­­­­­­­­­___________________________________________ ­­­­____/____/____

Name (please print)­­­­­ ___________________________________________________________

I consent to be audio recorded Yes/No

I would like to review a written form of my yarn/yarning questions Yes/No

I would like to receive an emailed copy of the report Yes/ No

Email (for those who would like a copy of the report) __________________@____________
