# Supplementary material for: The Marri Gudjaga project: a study protocol for a randomised control trial using Aboriginal peer support workers to promote breastfeeding of Aboriginal babies
Source: BMC Public Health. 2023 May 4;23:823. doi: 10.1186/s12889-023-15558-2 (PMC10161673; doi:10.1186/s12889-023-15558-2)
Supplement: Supplementary file 7 — Supplementary Material 7 [file 12889_2023_15558_MOESM7_ESM.pdf]

Consent - email version

Please complete the survey below.

Thank you!

The Marri-gudjaga project

You have been contacted as you expressed your interest in participating in our research project. You may have received a showbag from your local health centre providing information in relation to the project.

Your participation in project is is completely voluntary. This means that you do not have to participate unless you want to. We would like to ask you to complete a short survey. You will then receive another short survey around 6 weeks after your baby is born, then at 4 months and 6 months. You have elected to complete the surveys via email instead of over the phone. Then you will receive text message (or email if preferred) at 12 months.

Please contact @uow.edu.au or call 4221 5992 if you have any questions or issues

By providing your details below you are consenting to participate in the study.

I consent to participate in the research project.
◯ Yes
◯ No

First name: ______________________________

Surname (last name): ______________________________

Date of Birth: ______________________________
(enter as DD/MM/YYYY)

Could I please get your postal address as I would like to send you a $50 voucher after we complete the baseline survey: ______________________________

Please provide the best phone number to contact you on? ______________________________

If I can't catch you on this phone number is there another number I can get you on? (e.g. home phone number or additional mobile) ______________________________
(enter additional phone number)

Could you please confirm your email address we can use to contact you? ______________________________

Thank you for completing the consent form and for agreeing to participate in the project.

We will know ask you to complete a short survey.

Please use the link provided in the email.

Consent

Participant Code	______________________
My name is ____________. I am calling you regarding the Marri gudjada project supporting indigenous babies and mothers. You may have received a showbag from your local health centre providing information about the project.<br><br>The project involves a 5 min interview to start with which we can do now. We will then call you back 6 weeks after your baby is born, then at 4 months and 6 months. Each conversation will only be 5 mins again. Then you will receive text message at 12 months. Your participation in project is completely voluntary. This means that you do not have to take part unless you want to.<br><br>Do you have any questions?<br><br>Do you agree (consent) to participate in the project?	◯ Yes - I consent to participating in the project<br>◯ No - I do not consent to participate in the project
Date of consent:	______________________
First name:	______________________
Surname (last name):	______________________
Date of Birth:	______________________
Could I please get your postal address as I would like to send you a $50 voucher after we complete the 6 week survey:	______________________
(Enter in - do not ask of participant) Project site:	◯ Site 1<br>◯ Site 2<br>◯ Site 3<br>◯ Site 4<br>◯ Site 5<br>◯ Site 6<br>(Site 1 = ; Site 2 = ; Site 3 = ; Site 4 = ; Site 5 = ; Site 6 =)
Is this the best phone number to contact you on?	◯ Yes<br>◯ No
If I can't catch you on this phone number is there another number I can get you on? (e.g. home phone number or additional mobile)	______________________<br>(enter additional phone number)

Could you please provide an email address we can use to contact you?	__________________________________<br>(leave blank if no email)
(If under 18) As you are under 18 we will require consent from a parent or guardian. Are they present with you at the moment? (If yes) Could you please put them on the phone so I can ask them permission for you to be a part of the project?<br><br>(If parent present) - My name is (insert name of reseacher) and I am a researcher on the ** project. The project is looking at supporting indigenous mothers and their infants. The project will involve participants completing a few suverys over 12 months. Do you consent to (insert name) participating in the project?	◯ Yes<br>◯ No
(If under 18) Please provide a name of a parent or guardian:	__________________________________
(if under 18) Please provide a contact phone number for the parent or guardian:	__________________________________
I will now read a statement regarding future directions of the research project. Do you give permission to be contacted in the future regarding long term follow-up associated with this project?<br><br>Long term follow-up may include an SMS at 2 years such as "Are you still breastfeeding your child?"	◯ Yes<br>◯ No
Would you like to receive reports and findings of the project after its completion?	◯ Yes<br>◯ No
